# Leveraging a meta-learning approach to advance the accuracy of Na_v_ blocking peptides prediction

**DOI:** 10.1038/s41598-024-55160-z

**Published:** 2024-02-23

**Authors:** Watshara Shoombuatong, Nutta Homdee, Nalini Schaduangrat, Pramote Chumnanpuen

**Affiliations:** 1 Center for Research Innovation and Biomedical Informatics, Faculty of Medical Technology, Mahidol University, Bangkok, 10700 Thailand; 2 Department of Zoology, Faculty of Science, Kasetsart University, Bangkok, 10900 Thailand; 3 Omics Center for Agriculture, Bioresources, Food, and Health, Kasetsart University (OmiKU), Bangkok, 10900 Thailand

**Keywords:** Toxin, Sodium channel, Bioinformatics, Machine learning, Feature selection, Feature representation, Meta-learning, Computational biology and bioinformatics, Computational models, Machine learning

## Abstract

The voltage-gated sodium (Na_v_) channel is a crucial molecular component responsible for initiating and propagating action potentials. While the *α* subunit, forming the channel pore, plays a central role in this function, the complete physiological function of Na_v_ channels relies on crucial interactions between the α subunit and auxiliary proteins, known as protein–protein interactions (PPI). Na_v_ blocking peptides (NaBPs) have been recognized as a promising and alternative therapeutic agent for pain and itch. Although traditional experimental methods can precisely determine the effect and activity of NaBPs, they remain time-consuming and costly. Hence, machine learning (ML)-based methods that are capable of accurately contributing in silico prediction of NaBPs are highly desirable. In this study, we develop an innovative meta-learning-based NaBP prediction method (MetaNaBP). MetaNaBP generates new feature representations by employing a wide range of sequence-based feature descriptors that cover multiple perspectives, in combination with powerful ML algorithms. Then, these feature representations were optimized to identify informative features using a two-step feature selection method. Finally, the selected informative features were applied to develop the final meta-predictor. To the best of our knowledge, MetaNaBP is the first meta-predictor for NaBP prediction. Experimental results demonstrated that MetaNaBP achieved an accuracy of 0.948 and a Matthews correlation coefficient of 0.898 over the independent test dataset, which were 5.79% and 11.76% higher than the existing method. In addition, the discriminative power of our feature representations surpassed that of conventional feature descriptors over both the training and independent test datasets. We anticipate that MetaNaBP will be exploited for the large-scale prediction and analysis of NaBPs to narrow down the potential NaBPs.

## Introduction

Voltage-gated sodium (Na_v_) channels play a role in both physiological and pathophysiological electrical signalling in nerve and muscle cells^1^. This channel serves as a vital molecular element in initiating and propagating action potentials. Although the α subunit, which constitutes the channel pore, is pivotal in this process, the complete physiological activity of Nav channels relies on crucial interactions between the α subunit and auxiliary proteins, known as protein–protein interactions (PPI)^2^. The activity of voltage-gated sodium channels has been linked to numerous diseases, encompassing epilepsy, chronic pain, cardiovascular disorders, cancers, immune system conditions, as well as neuromuscular and respiratory disorders^3^. Moreover, voltage-gated sodium channels (VGSCs) consist of a pore-forming α subunit and additional subunits, arranged into four operational voltage-sensor domains (VSD1-4). The substantial therapeutic potential linked to Na_v_s has been thoroughly investigated in prior studies and remains a topic of keen interest in current research^4–6^.

In general, voltage-gated sodium channels trigger action potentials in cells that are electrically excitable by increasing sodium permeability. They additionally function as molecular targets for neurotoxins, which bind to various receptor sites, thereby modifying voltage-dependent activation, conductance, and inactivation^7^. Among the various types of ion channels, VGSCs play a vital role in the initiation and transmission of action potentials, encoding the responses of nociceptors to stimuli^8^. Thus, considered as prime targets for pain therapeutics, the VGSCs hold significant potential in their development^9^. The immense potential of proteomics and other OMICS technologies in this field enables highly accurate detection and quantification of bioactive peptides that specifically and potently block established therapeutic targets, including voltage-gated sodium channels^10–12^. Many peptides derived from the venom of spiders, snails, sea anemone, and other animals exhibit remarkable potency and selectivity against voltage-gated sodium channels (Na_v_s)^10,11,13–17^. Acting as promising lead compounds, these peptides contribute to the development of antiepileptic, antiarrhythmic drugs, and analgesics, serving as valuable pharmacological tools^18^. Classified based on their mechanism of action, Na_v_ blocking peptides (NaBPs) can be categorized into two groups. The first group consists of pore blockers that obstruct the passage of Na + by engaging with the channel vestibule. The second group, known as 'gating modulators' or allosteric modulators, interacts with one or more voltage-sensor domains to impact the gating and kinetics of the channel^18^. Although Na_v_s show their significant value as potential drug targets, their therapeutic properties has not yet been fully realized^19^. Therefore, computational or machine learning (ML)-based methods are highly desirable to perform the high-throughput prediction and analysis of NaBPs using sequence information only.

Previously, sequence-based computational tools such as PPLK^+^C^20^, NTXpred^21^, and PEP-PRED^Na+^^3^ were developed specifically to predict toxins capable of blocking ion channels. To be specific, PPLK^+^C, NTXpred, and PEP-PRED^Na+^ were capable of predicting peptides that block potassium, sodium, and Na_v_ channels, respectively. To the best of our knowledge, PEP-PRED^Na+^ is the only computational tool that can predict peptides blocking Na_v_ channels. This method was constructed using the random forest algorithm in combination with ten selected physicochemical properties (AAindex). PEP-PRED^Na+^ achieved cross-validation and independent test Matthew's correlation coefficient (MCC) values of 0.670 and 0.840, respectively. Although this method achieved great performance over the independent test dataset, it still has some limitations that need to be addressed. Firstly, the exploration of other conventional feature descriptors was not implemented. Secondly, because the existing method was developed using a single ML-based approach, its discriminative power might not be robust in some cases. This implies that the performance of the ML-based methods of NaBP prediction might be limited in terms of their classification ability. Lastly, the prediction performance of the existing method was still unsatisfactory.

In this study, we propose a novel and high-accuracy NaBP predictor using a meta-learning approach (MetaNaBP). The major advantages and contributions of MetaNaBP over other related ML models and exiting methods can be summarized as follows: (i) MetaNaBP is the first meta-learning model designed for NaBPs prediction. Specifically, we evaluated and investigated a wide range of sequence-based feature descriptors containing multiple perspectives of sequence information, cooperating with six different ML algorithms (i.e., random forest (RF), extreme gradient boosting (XGB), support vector machine (SVM), partial least squares (PLS), multi-layer perceptron (MLP), and k-nearest neighbor (KNN)) to create new feature representations containing class and probabilistic information. Then, these feature representations were optimized using a two-step feature selection method, and the best feature set was selected to develop the stable meta-predictor; (ii) Comparative results with PEP-PRED^Na+^ on the independent test dataset showed that MetaNaBP was capable of increasing MCC from 0.820 to 0.898, accuracy (ACC) from 0.890 to 0.948, sensitivity (SN) from 0.900 to 0.979, indicating that MetaNaBP could be a useful tool to precisely identify NaBPs; and (iii) The performance comparison among several feature descriptors was sufficient to demonstrate that our feature representations provided the most discriminative power for identifying NaBPs.

## Materials and methods

### Dataset construction and preprocessing

To ensure a fair comparison between the existing method and our proposed method, we utilized the same benchmark dataset generated by the previous work^3^. This dataset was originally extracted from NTXpred, created by Saha and Raghava^21^. It comprises 244 NaBPs and 244 non-NaBPs, with 240 positive samples and 236 negative samples remaining after excluding samples containing special symbols. From these samples, 80% of the benchmark dataset were randomly selected to construct the training dataset (i.e., 192 positive samples and 188 negative samples), whereas the remaining samples were used to construct the independent test dataset (i.e., 48 positive samples and 48 negative samples).

### Feature representation

Here, we employed fifteen types of feature descriptors using eight different feature encoding schemes to fully capture the useful patterns of NaBPs (Table 1). These feature descriptors have previously demonstrated reasonable prediction performance for several protein properties^22–29^. They are derived from three groups of feature descriptors, including sequence composition-based, physicochemical property-based, and pseudo-amino acid composition-based features. A detailed description of three groups is summarized as follows.

**Table 1:** Summary of eight different feature encoding algorithms along with their corresponding description and dimension.

Descriptors^a^	Description	Dimension	Reference
AAC	Frequency of 20 amino acids	20	^22,25,26^
CTD	Composition, transition and distribution	273	^22,25,26^
CTDC	Percentage of particular amino acid property groups	21	^25,26^
CTDD	Percentage of mutual conversion in amino acid properties	21	^25,26^
CTDT	Distribution of amino acid properties in sequences	105	^25,26^
DPC	Frequency of 400 dipeptides	400	^22,25^
PAAC	Pseudo amino acid composition	20 + λ	^25,26^
TPC	Frequency of 8000 dipeptides	8000	^22,25,26^

^a^AAC: amino acid composition, CTD: composition translation and distribution, CTDC: CTD composition, CTDT: CTD distribution, CTDT: CTD transition, DPC: dipeptide composition, PAAC: pseudo amino acid composition, TPC: tripeptide composition.

For the sequence composition-based group, we applied three well-known feature encodings, including AAC, DPC, and TPC^30–32^. AAC encoding calculates the occurrence frequencies of the 20 possible amino acids. The composition of the *i*th amino acid ($aa\left(i\right)$) can be defined as:

1 $$aa\left(i\right)=\frac{{AA}_{i}}{L}$$

where ${AA}_{i}$ is the number of the *i*th amino acid, and *L* is the length of the query protein. Meanwhile, DPC and TPC encodings calculate the occurrence frequencies of one and two consecutive amino acids, respectively. The composition of the *j*th dipeptide ($dp\left(j\right)$) and *k*th tripeptide ($tp\left(k\right)$) can be defined as:

2 $$dp\left(j\right)=\frac{{DP}_{j}}{L-1}$$

3 $$tp\left(k\right)=\frac{{TP}_{k}}{L-2}$$

where ${DP}_{j}$ and ${TP}_{k}$ are the number of the *j*th dipeptide and *k*th tripeptide, respectively. As a result, AAC, DPC, and TPC encoding generate 20-D, 400-D, and 8000-D feature vectors, respectively.

CTD encoding involves three feature descriptors (i.e., composition (C), transition (T) and distribution (D))^33^. This encoding is naturally used to characterize the global distribution patterns and physicochemical properties of amino acids in the protein sequences. In particular, CTD groups 20 amino acids into 3 major groups based on seven physicochemical properties of amino acids, as summarized in Table S1.

For a given the *i*th physicochemical property (${PCP}_{i}$), CTDC represents the global percentage of each group of 20 amino acids, which can be calculated as follows:

4 $$CTDC=(\frac{{N}_{PCP1,G1}}{L}, \frac{{N}_{PCP1,G2}}{L},\frac{{N}_{PCP1,G3}}{L},\dots ,\frac{{N}_{PCP7,G1}}{L}, \frac{{N}_{PCP7,G2}}{L},\frac{{N}_{PCP7,G3}}{L})$$

where ${N}_{PCP1,G1}$ is the number of amino acids involved in Group #1 of physicochemical property #1, with physicochemical property #1 being hydrophobicity (Table S1). Using CTDC, any protein sequence can be encoded as a 21-D feature vector. CTDT encoding computes the number of amino acids in the current group followed by amino acids of another group, while CTDD encoding calculates the distribution descriptor involving the fractions of the entire protein sequence. Thus, the dimensions of the CTDT and CTDD feature vectors are 21 and 105, respectively.

In case of PAAC encoding^34^, it was initially introduced to address the issue of sequence-order information. In particular, the PAAC feature vector can be represented as:

5 $$P=\left[\begin{array}{c}\begin{array}{c}{p}_{1}\\ {p}_{2}\end{array}\\ \begin{array}{c}\begin{array}{c}\dots \\ {p}_{20}\end{array}\\ \begin{array}{c}\begin{array}{c}{p}_{20+1}\\ \dots \end{array}\\ {p}_{20+\lambda }\end{array}\end{array}\end{array}\right]$$

6 $${p}_{c}=\frac{{f}_{c}}{{\sum }_{c=1}^{20}{f}_{c}+\omega {\sum }_{j}^{\lambda }{\theta }_{j} } (1<c<20)$$

7 $${p}_{c}=\frac{\omega {\theta }_{c-20}}{{\sum }_{c=1}^{20}{f}_{c}+\omega {\sum }_{j=1}^{\lambda }{\theta }_{j} } (21<c<20+\lambda )$$

8 $${\theta }_{j}=\frac{1}{L-\lambda }\sum_{i=1}^{N-1}\Theta ({P(S}_{i}),{P(S}_{i+j}))$$

where ${f}_{c}$ is the percentage composition of the amino acid type c, while ${\theta }_{j}$ represents the jth rank of $\Theta ({P(S}_{i}),{P(S}_{i+j}))$ and $\lambda$ is the maximum correlation length. As a result, the dimension of the PAAC feature vector is 21 + $\lambda$, respectively, where $\lambda$ = 1, 2, 3, …, 8. All the fifteen types of feature descriptors were extracted using the protr package in the R programming language^35^.

### Machine learning algorithms

In this work, six well-known ML algorithms were applied to develop the proposed model, including random forest (RF), extreme gradient boosting (XGB), support vector machine (SVM), partial least squares (PLS), multi-layer perceptron (MLP), and k-nearest neighbor (KNN). The detailed implementations and parameter optimizations of these six ML models are briefly summarized below.

RF is considered a powerful ensemble learning model that makes the final prediction output based on the voting strategy of multiple decision trees^36^. Two essential parameters in the development of RF models are the number of randomly selected features (*mtry*) and decision trees (*ntree*). The search range for *mtry* and *ntree* were {3, 5, 7, 9, 10} and {20, 50, 100, 200, 300}, respectively. We trained and optimized RF model using the *randomForest* package in the R language. XGB has been shown to achieve reasonable accuracy in many machine learning problems^37–39^. The basic idea of XGB is to create a strong prediction model by combining several weak prediction models. More detailed information on XGB is provided in the study by Chen and Carlos^40^. Herein, the XGB model was trained and optimized using the *xgboost* package in R language. The details of search ranges of XGB’ parameters are summarized in Table S2. For SVM, it is well-recognized as an efficient ML algorithm for binary classification tasks. The principle idea of SVM is to determine an optimal hyperplane that can maximize the margin between two classes (i.e., positive and negative samples). The SVM model was trained based on the cost parameter ({0.25, 0.5, 1, 2, 4}) using the *e1071* package in the R language. Detailed information on MLP, PLS, and KNN is summarized our previous studies^25,26,32^.

### Meta-learning framework of MetaNaBP

**Figure 1:**
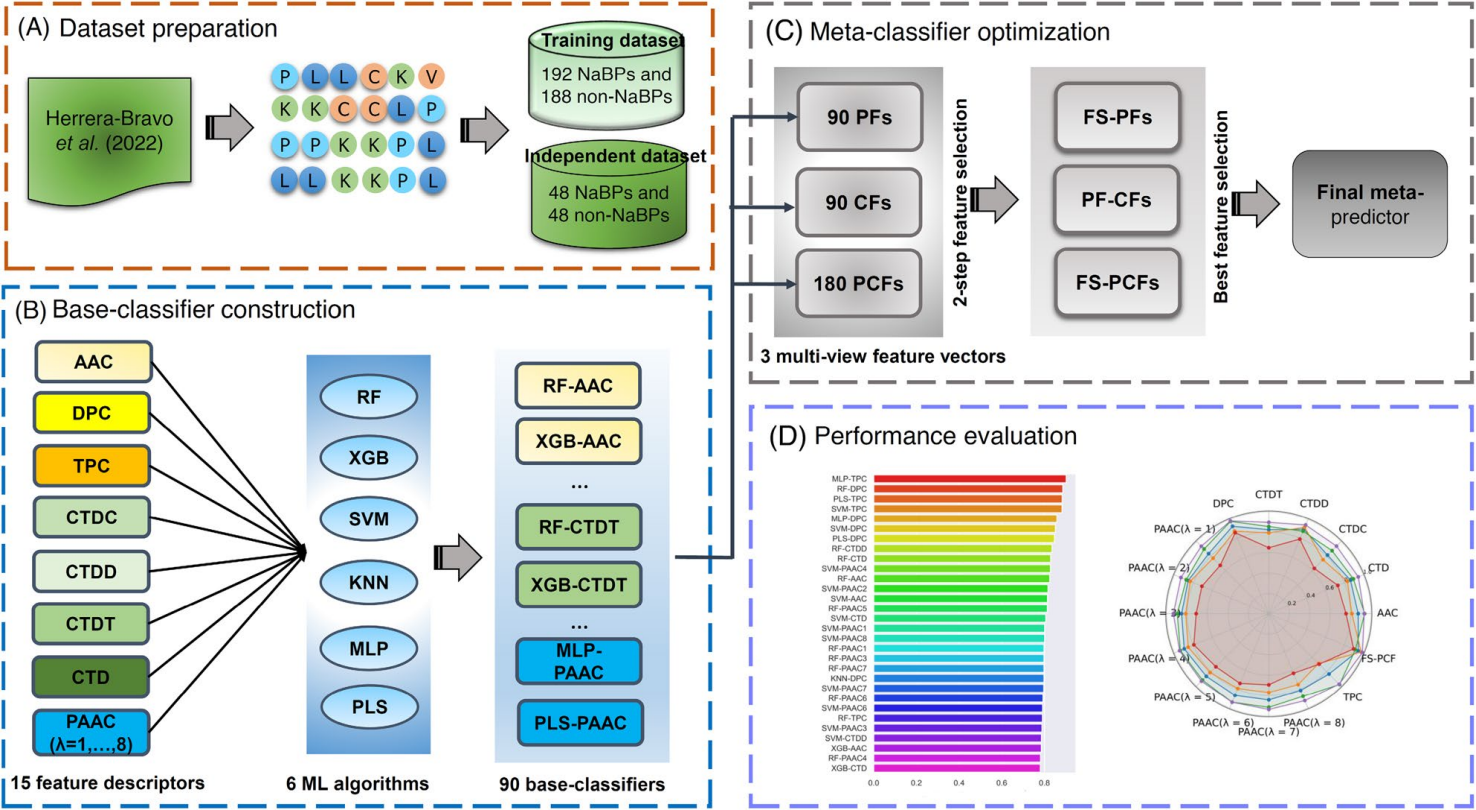
The framework of the proposed MetaNaBP based on a meta-learning approach. There exist four main steps, including (**A**) dataset preparation. (**B**) base-classifier construction. (**C**) meta-classifier optimization. (**D**) performance evaluation.

In this study, MetaNaBP is a meta-learning approach developed to enhance the prediction of NaBPs. To date, the meta-learning approach has been efficiently used for the prediction and analysis of various peptides^22–24^ and drugs^41–43^. As illustrated in Fig. 1, the meta-learning process involves two main steps: (i) base-classifier construction and (ii) meta-classifier optimization^22,23,32,44^.

In the first step, fifteen types of feature descriptors from eight different feature encoding were applied to represent NaBPs. These feature descriptors were individually inputted into six different ML algorithms. As a result, we obtained a total of 90 base-classifiers. During model training, all the base-classifiers were built and optimized using a grid search and the tenfold cross-validation approach. Detailed information regarding the search ranges of ML classifiers’ parameters are recorded in Table S2. In the next step, we utilized all the 90 base-classifiers to generate the estimated probabilities to be NaPBs and then obtained a 90-dimensional (90-D) feature vector containing 90 probabilistic features (PFs). For a given protein sequence *P*, its PFs are defined as follows:

9 $${\text{PFs}}=\left[{{\text{P}}({\text{M}}}_{1}, {{\text{FD}}}_{1}\right),{{\text{P}}({\text{M}}}_{1}, {{\text{FD}}}_{2}),{{\text{P}}({\text{M}}}_{1}, {{\text{FD}}}_{3})\dots .,{{\text{P}}({\text{M}}}_{{\text{i}}}, {{\text{FD}}}_{{\text{j}}}),\dots ,{{\text{P}}({\text{M}}}_{6}, {{\text{FD}}}_{15})]$$

where ${{\text{P}}({\text{M}}}_{{\text{i}}}, {{\text{FD}}}_{{\text{j}}})$ is the estimated probability derived from the i^th^ ML algorithm and the j^th^ feature descriptor. In general, each base-classifier is capable of providing two important pieces of information: estimated probabilities and classes to be NaPBs. In case of class information, it can be can represented by

10 $${\text{CFs}}=\left[{{\text{C}}({\text{M}}}_{1}, {{\text{FD}}}_{1}\right),{{\text{C}}({\text{M}}}_{1}, {{\text{FD}}}_{2}),{{\text{C}}({\text{M}}}_{1}, {{\text{FD}}}_{3})\dots .,{{\text{C}}({\text{M}}}_{{\text{i}}}, {{\text{FD}}}_{{\text{j}}}),\dots ,{{\text{C}}({\text{M}}}_{6}, {{\text{FD}}}_{15})]$$

11 $${{\text{C}}({\text{M}}}_{{\text{i}}}, {{\text{FD}}}_{{\text{j}}})=\left\{\begin{array}{cc}1& ,{{\text{P}}({\text{M}}}_{{\text{i}}}, {{\text{FD}}}_{{\text{j}}})\ge 0.5\\ 0& ,{{\text{P}}({\text{M}}}_{{\text{i}}}, {{\text{FD}}}_{{\text{j}}})<0.5\end{array}\right.$$

where ${{\text{C}}({\text{M}}}_{{\text{i}}}, {{\text{FD}}}_{{\text{j}}})$ is the estimated class label derived from the *i*th ML algorithm and the *j*th feature descriptor. As a result, we obtained a 90-D feature vector containing 90 CFs. In this study, we fused them to construct a multi-view feature vector (PCF) containing 90 PFs and 90 CFs. To enhance the feature ability, a two-step feature selection approach was applied to optimize the PF, CF, and PCF feature vectors. In brief, the first step is to calculate the importance score of each feature using the mean decrease of Gini index (MDGI) and then generate the ranked features in descending order. The second step is to identify the optimal feature set using the sequential forward search (SFS) strategy. In SFS, we construct several feature sets containing *m* top-ranked importance features, where *m* = 5, 10, 15, …, *n*. After that, we sequentially input *m* top-ranked importance features into RF classifiers. In this study, the feature sets is considered the best one when the corresponding RF classifiers provide the highest MCC. The best feature sets of PF, CF, and PCF are referred as FS-PF, FS-CF, and FS-PCF, respectively.

### Performance measurement

We used five well-known performance metrics to assess the prediction performance of the prediction models, including ACC, MCC, SN, F1, specificity (SP), the area under the receiver operating characteristics (ROC) curve (AUC), and precision (PRE)^45–47^. ACC, MCC, SN, and SP are defined as:

12 $${\text{SN}}=\frac{{\text{TP}}}{\left({\text{TP}}+{\text{FN}}\right)}$$

13 $${\text{SP}}=\frac{{\text{TN}}}{\left({\text{TN}}+{\text{FP}}\right)}$$

14 $${\text{ACC}}=\frac{{\text{TP}}+{\text{TN}}}{\left({\text{TP}}+{\text{TN}}+{\text{FP}}+{\text{FN}}\right)}$$

15 $${\text{MCC}}=\frac{{\text{TP}}\times {\text{TN}}-{\text{FP}}\times {\text{FN}}}{\sqrt[]{({\text{TP}}+{\text{FP}})({\text{TP}}+{\text{FN}})({\text{TN}}+{\text{FP}})({\text{TN}}+{\text{FN}})}}$$

16 $${\text{F}}1=2\times \frac{{\text{TP}}}{2{\text{TP}}+{\text{FP}}+{\text{FN}}}$$

17 $${\text{PRE}}=\frac{{\text{TP}}}{{\text{TP}}+{\text{FP}}}$$

In this study, the numbers of correctly predicted NaBPs and non-NaBPs are denoted as TP and TN, respectively. Additionally, the number of non-NaBPs predicted as NaBPs is denoted as FP, while the number of NaBPs predicted as non-NaBPs is denoted as FN^48–50^.

## Results and discussion

### ***Analysis of amino acid preference of Na***_***v***_***s blocking peptides***

**Figure 2:**
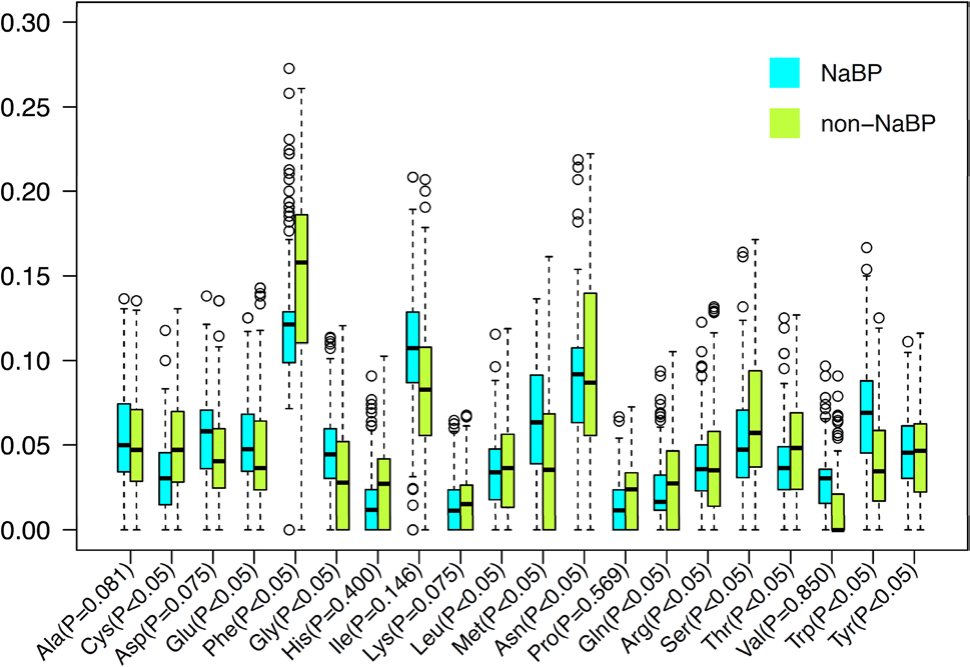
Boxplots of amino acid compositions of 20 amino acids for NaBPs and non-NaBPs. X- and Y-axes represent 20 amino acids along with their *p*-value.

We employed a Student’s *t*-test to assess the statistically significant difference in amino acid preference between NaBPs and non-NaBPs, as summarized in Table 2. Interestingly, hydrophobic amino acid residues (i.e., Tyr, Ala, Trp, and Gly) and negatively charge amino acids (Asp and Glu) significantly dominated in NaBPs with a *p*-value of less than 0.05 (Fig. 2). Among these nonpolar side-chain residues, Try and Gly showed the highest difference scores of 0.026 and 0.025, respectively. On the other hand, hydrophilic or polar uncharged residues (Cys, Ser, Gln, and Thr) and positively charged side-chain amino acids (Arg and Lys) were more preferable in non-NaBPs.

**Table 2:** Average amino acid compositions of Na_v_ blocking (Positive) and non-Na_v_ (Negative) blocking peptides along with difference values and *p*-value.

Amino acid	Positive	Negative	Difference	*p*-value
A-Ala	0.055	0.050	0.005	< 0.05
C-Cys	0.126	0.149	−0.023	< 0.05
D-Asp	0.053	0.048	0.006	0.075
E-Glu	0.043	0.036	0.008	< 0.05
F-Phe	0.022	0.029	−0.006	< 0.05
G-Gly	0.110	0.085	0.025	< 0.05
H-His	0.014	0.015	−0.001	0.400
I-Ile	0.036	0.039	−0.004	0.149
K-Lys	0.090	0.097	−0.007	0.075
L-Leu	0.064	0.045	0.019	< 0.05
M-Met	0.013	0.023	−0.009	< 0.05
N-Asn	0.055	0.046	0.009	< 0.05
P-Pro	0.041	0.043	−0.002	0.569
Q-Gln	0.016	0.029	−0.013	< 0.05
R-Arg	0.032	0.054	−0.021	< 0.05
S-Ser	0.051	0.068	−0.017	< 0.05
T-Thr	0.039	0.051	−0.012	< 0.05
V-Val	0.044	0.044	0.000	0.850
W-Trp	0.030	0.013	0.017	< 0.05
Y-Tyr	0.066	0.040	0.026	< 0.05

A similar pattern of the hydrophobic and negatively charged portions of Na_v_ inhibitors were also observed in domain IV voltage sensor-targeting peptides^51^. For instance, the conserved hydrophobic patch surrounded by a charged ring was typically observed in GMTs isolated from spiders. According to experiments with hydrophobic substitution analogs of tarantula peptide GpTx-1, the lower hydrophobicity of NaBPs could lead to a significant decrease in potency on Na_v_ voltage sensor binding ability^5,51^. This structural feature has been proposed to promote not only NaBPs–voltage-gated ion channel interactions but also NaBPs–lipid membrane interactions^15^. Typically, increasing the hydrophobicity at specific amino acid positions within the peptide, presumed to directly interact with the channel, generally resulted in the maintenance or augmentation of the potency of domain IV voltage sensors in Na_v_1.4 and Na_v_1.7^6,52^. Moreover, only van der Waals force and hydrophobic interactions to the compound was provided by the lipid-exposed pocket. Dealing with the effects of drug binding, deglycosylation, and the role of hydrophobic residues in the voltage sensors, it could be implied that side-chain hydrophobicity might be important to the interaction of NaBPs with the voltage sensor domains. It has been widely recognized that a shared characteristic among voltage-gated ion channels is the presence of voltage sensors. Positively charged residues (Arg and Lys) are interspersed every three amino acids within the S4 transmembrane segments, separated by two non-polar residues^53^. This basic feature of the region makes the fragments of structure with an acidic binding property easily attach to it. Thus, negatively charged side chains (Asp and Glu) might be required for the interaction of NaBPs with the S4s segment.

Focusing on the aromatic side-chain residues, both Trp and Tyr were significantly dominated in NaBPs with a *p*-value of less than 0.05. These pieces of evidence might be correlated with the fact that the conformations of the aromatic groups of Trp and Tyr are prone to forming π-π stacking interaction, binding to the selectivity pocket of the voltage sensor domains. Similar to the conserved aromatic residue in NaBPs isolated from scorpion, Trp is crucial for the interaction with sodium channels Na_v_1.7, Na_v_1.4 and Na_v_1.5^54^.

### Performance evaluation of different feature encoding methods and ML algorithms

**Figure 3:**
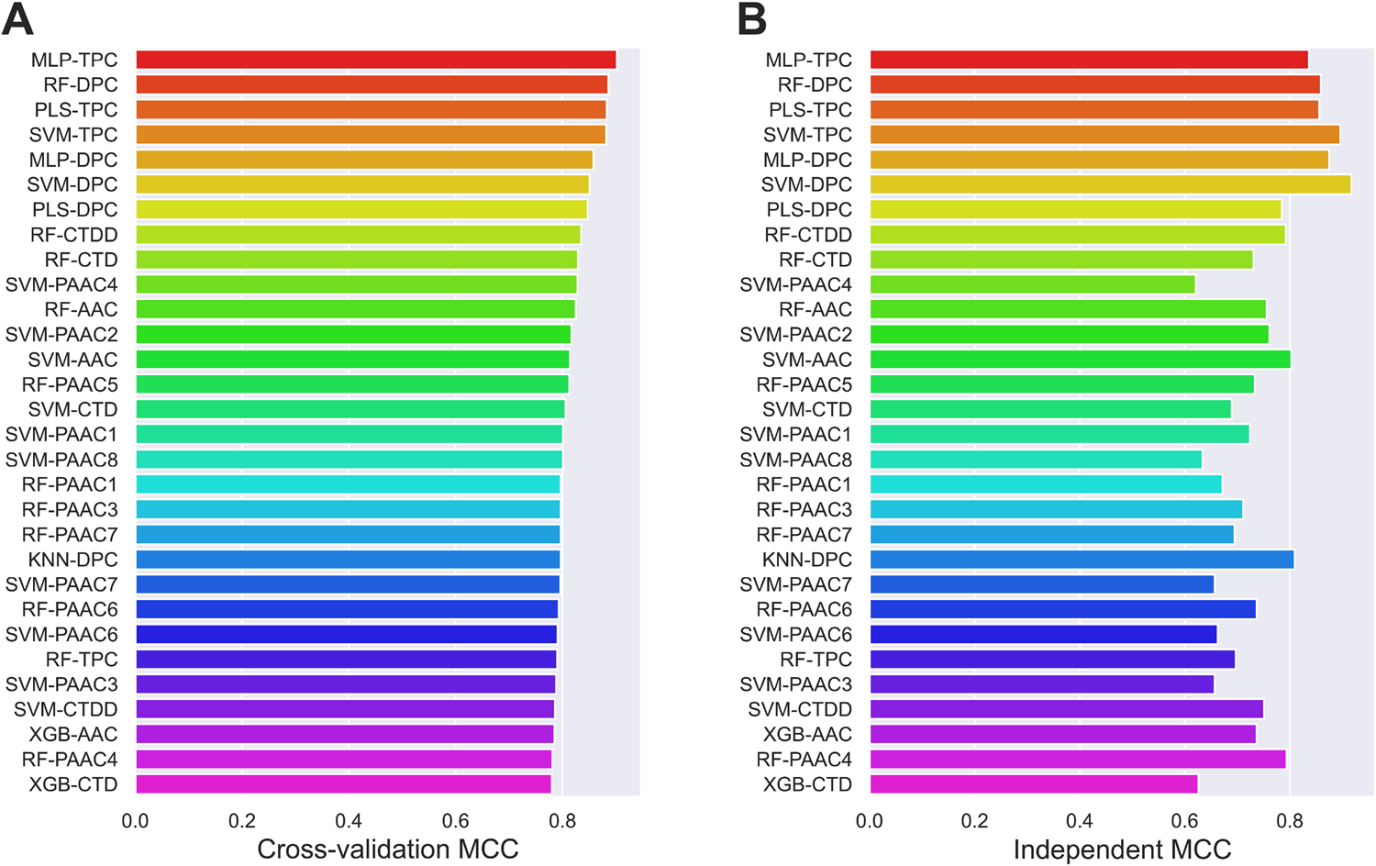
MCC values of top-30 ML classifiers as evaluated using the training (**A**) and independent (**B**) datasets, where PAACi represents PAAC (*λ* = *i*).

In this section, we used five performance metrics to compare the impact of different feature encoding methods and ML algorithms on the prediction of NaBPs. Each developed ML classifier was evaluated using both the tenfold cross-validation and independent tests. After that, we selected the top-five ML classifiers with the highest cross-validation MCC for conducting the comparative analysis. The results of all the ML classifiers over the tenfold cross-validation and independent tests are recorded in Fig. 3 along with Fig. S1 and Tables S3-S4, while the results of the top-ten ML classifiers are documented in Table S5. Based on the cross-validation MCC, the top-ten ML classifiers consisted of MLP-TPC, RF-DPC, PLS-TPC, SVM-TPC, MLP-DPC, SVM-DPC, PLS-DPC, RF-CTDD, RF-CTD, and SVM-PAAC($\lambda$ = 4), with corresponding MCCs of 0.902, 0.886, 0.883, 0.882, 0.858, 0.851, 0.847, 0.835, 0.829, and 0.828. Interestingly, seven out of the top-ten ML classifiers were developed based on DPC and TPC encoding methods. This indicates that DPC and TPC might be important for effectively capturing information about NaBPs. To demonstrate this point, we calculate average five performance metrics for fifteen types of feature descriptors over six ML algorithms (Table S6). The top-five feature descriptors, having the highest average MCC of 0.830, 0.795, 0.751, 0.750, and 0.747, were DPC, TPC, PAAC($\lambda$ = 7), PAAC($\lambda$ = 6), and AAC, respectively. As shown in Fig. 3 and Table S5, MLP-TPC is considered the best among ML classifiers developed in this study, achieving ACC of 0.950, SN of 0.917, SP of 0.984, and AUC of 0.988 on the training dataset. On the other hand, for the independent test dataset, the best ML classifier was SVM-DPC, achieving ACC, SN, SP, MCC, and AUC of 0.958, 0.938, 0.979, 0.917, and 0.988, respectively. In brief, the ranks of MLP-TPC were 1 and 6 as evidenced by both the tenfold cross-validation and independent test, respectively. This confirms that using single ML-based approaches could not provide robust and stable prediction performance.

### Development of MetaNaBP

**Figure 4:**
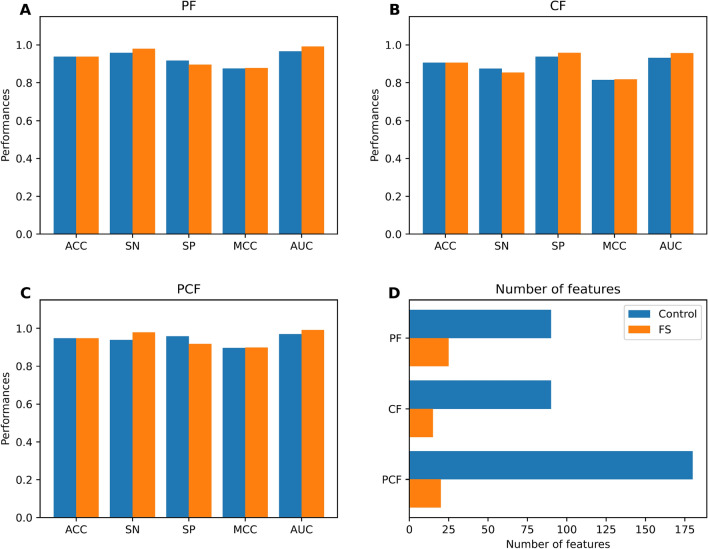
Performance comparison of different feature representations over the independent test dataset. (**A-C**) The performances of different feature representations in terms of ACC, SN, SP, MCC and AUC. (**D**) The feature number of different feature representations.

To overcome the limitation of single ML-based approaches, we employed several types of feature representations (i.e. PF, CF, PCF, FS-PF, FS-CF, and FS-PCF) to develop different meta-classifiers. Table 3 and Fig. 4 shows that PF and PCF attain similar and better performance compared with CF in terms of MCC. Specifically, PF and PCF exhibited ACC, SN, and MCC, and AUC with ranges of 0.966–0.982, 0.943–0.964, and 0.933–0.964, respectively, on the training dataset. To improve the prediction performance of the model and reduce computational time, we employed a two-step feature selection strategy. As seen in Table 3, the MCC of FS-PF and FS-PCF is better than their controls in terms of the tenfold cross-validation test. Among several types of our feature representations, it can be stated that FS-PCF outperformed other fusion features in terms of cross-validation MCC. Regarding the results of the independent test, FS-PCF secures the best prediction performance in terms of ACC (0.948), SN (0.979), and MCC (0.898) (Fig. 4). Altogether, we selected FS-PCF as the best feature representation and inputted it into the RF classifier to develop the final meta-classifier, namely MetaNaBP.

**Table 3:** Cross-validation and independent test results of different feature representations.

Evaluation strategy	Feature	Number of features	ACC	SN	SP	MCC	AUC
Cross-validation	PF	90	0.982	0.964	**1.000**	0.964	**0.996**
	CF	90	0.947	0.911	0.984	0.897	0.966
	PCF	180	0.966	0.943	0.989	0.933	**0.996**
	FS-PF	25	0.984	0.969	**1.000**	0.969	0.992
	FS-CF	15	0.942	0.901	0.984	0.887	0.956
	FS-PCF	20	**0.987**	**0.974**	**1.000**	**0.974**	0.991
Independent test	PF	90	0.938	0.958	0.917	0.876	0.965
	CF	90	0.906	0.875	0.938	0.814	0.931
	PCF	180	**0.948**	0.938	**0.958**	0.896	0.970
	FS-PF	25	0.938	**0.979**	0.896	0.878	0.992
	FS-CF	15	0.906	0.854	**0.958**	0.817	0.956
	FS-PCF	20	**0.948**	**0.979**	0.917	**0.898**	**0.991**

The best performance value for each performance metrics across different feature representations is highlighted in bold.

### Meta-learning can improve the effectiveness and generalization ability

To reveal the effectiveness of the meta-learning approach, assessed and compared the performance of MetaNaBP and other base-classifiers using five performance metrics, on both the training and independent datasets. For convenient of comparative analysis, the performance of MetaNaBP was compared against the top-five base-classifiers, including MLP-TPC, RF-DPC, PLS-TPC, SVM-TPC, and MLP-DPC (Table 4). Impressively, for both the cross-validation and independent test, MetaNaBP demonstrated better performance in terms of ACC, MCC, AUC, and SP compared to the top-five base-classifiers. Moreover, MCC values of MetaNaBP were 7.19% and 6.13% higher than the best base-classifier (i.e., MLP-TPC) on the training and independent dataset, respectively.

**Table 4:** Performance comparison of MetaNaBP and top-five powerful prediction models on the training and independent test datasets.

Evaluation strategy	Method	ACC	SN	SP	MCC	AUC
Cross-validation	MLP-DPC	0.929	0.927	0.931	0.858	0.971
	SVM-TPC	0.939	0.901	0.979	0.882	0.988
	PLS-TPC	0.939	0.896	0.984	0.883	0.989
	RF-DPC	0.939	0.880	**1.000**	0.886	0.977
	MLP-TPC	0.950	0.917	0.984	0.902	0.988
	MetaNaBP	**0.987**	**0.974**	**1.000**	**0.974**	**0.991**
Independent test	MLP-DPC	0.938	0.938	0.938	0.875	0.973
	SVM-TPC	**0.948**	0.938	0.958	0.896	0.985
	PLS-TPC	0.927	0.896	0.958	0.856	0.989
	RF-DPC	0.927	0.875	**0.979**	0.859	0.984
	MLP-TPC	0.917	0.875	0.958	0.836	0.987
	MetaNaBP	**0.948**	**0.979**	0.917	**0.898**	**0.991**

The best performance value for each performance metrics across different methods is highlighted in bold.

**Figure 5:**
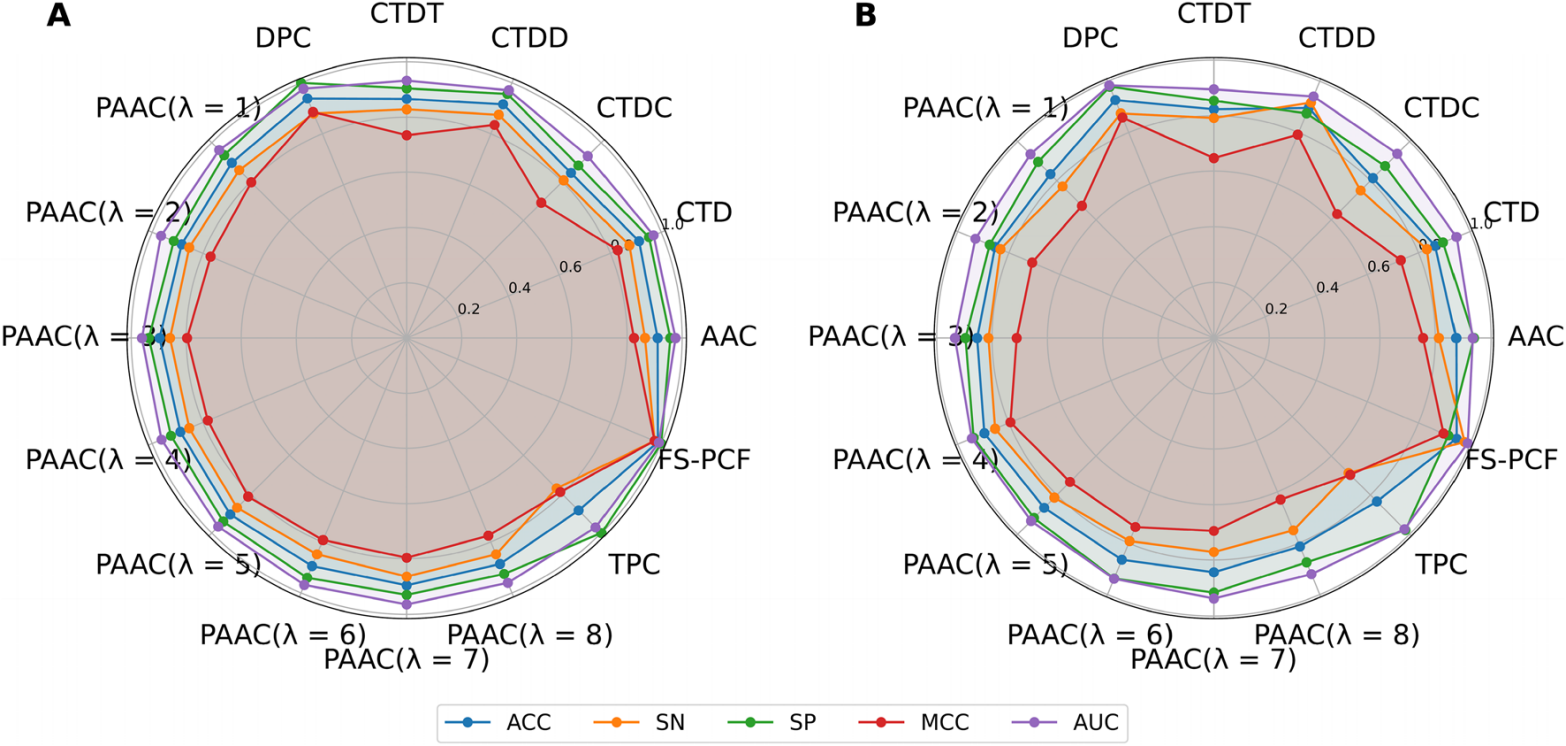
ACC, SN, SP, MCC, and AUC values of FS-PCF and conventional feature descriptors as evaluated by the tenfold cross-validation (**A**) and independent tests (**B**).

In addition, we investigated the feature ability of FS-PCF by comparing its performance against conventional feature descriptors. To make a fair test, we trained 15 individual RF models in conjunction with each type of feature descriptors and evaluate their performance. Summarized in Fig. 5 and Table 5, along with Table S7, are the detailed comparative results in terms of the tenfold cross-validation and independent tests. As visualized in Fig. 5, FS-PCF attained outstanding performance on four metrics (i.e., AUC, SN, ACC, and MCC) as observed in both the tenfold cross-validation and independent tests. Intriguingly, when compared with top-three conventional feature descriptors (i.e., DPC, CTDD, and CTD) over the independent test, FS-PCF was more effective and demonstrated an excellent ability in identifying NaBPs, achieving ACC of 0.948, SN of 0.979, MCC of 0.898, and AUC of 0.991 (Table 5). The improved performance of FS-PCF on the training dataset suggested that our feature representations can advance the efficiency of model training. Altogether, the comparative findings demonstrated that MetaNaBP was capable of precisely identifying NaBPs compared to its base-classifiers.

**Table 5:** Performance comparison of our feature (FS-PCF) and conventional feature descriptors on the independent test dataset.

Feature	ACC	SN	SP	MCC	AUC
AAC	0.875	0.813	0.938	0.756	0.934
CTD	0.865	0.833	0.896	0.731	0.948
CTDC	0.813	0.750	0.875	0.630	0.937
CTDD	0.896	0.917	0.875	0.792	0.941
CTDT	0.823	0.792	0.854	0.647	0.895
DPC	0.927	0.875	**0.979**	0.859	0.984
PAAC(λ = 1)	0.833	0.771	0.896	0.672	0.935
PAAC(λ = 2)	0.854	0.833	0.875	0.709	0.931
PAAC(λ = 3)	0.854	0.813	0.896	0.711	0.934
PAAC(λ = 4)	0.896	0.854	0.938	0.794	0.945
PAAC(λ = 5)	0.865	0.813	0.917	0.733	0.932
PAAC(λ = 6)	0.865	0.792	0.938	0.737	0.939
PAAC(λ = 7)	0.844	0.771	0.917	0.695	0.938
PAAC(λ = 8)	0.813	0.750	0.875	0.630	0.921
TPC	0.833	0.688	**0.979**	0.697	0.974
FS-PCF (Our feature)	**0.948**	**0.979**	0.917	**0.898**	**0.991**

The best performance value for each performance metrics across different methods is highlighted in bold.

### Comparison of MetaNaBP with the existing method

To assess the predictive efficacy of our proposed method, we compared the results of tenfold cross-validation and independent tests for MetaNaBP with those of PEP-PREDNa + ^3^. As seen in Table 6, it’s worth noting that there are five different prediction models (i.e., ET, RF, XGB classifier (XGBC), XGBRF classifier (XGBRFC), LightGBM classifier (LGBMC)) found in PEP-PRED^Na+^. Please note that the experimental results of these five different prediction models were directly obtained from the previous work^3^ to ensure a fair comparison. As can be seen, amongst the five different prediction models found in PEP-PRED^Na+^, LGBMC performed better that the others, as indicated by the tenfold cross-validation results, achieving an ACC of 0.840, SN of 0.840, SP of 0.850, F1 of 0.840, and MCC of 0.680.

**Table 6:** Performance comparison of MetaNaBP and the existing methods on the training and independent test datasets.

Evaluation strategy	Method	ACC	SN	SP	MCC	PRE	F1
Cross-validation	PEP-PRED^Na+^ (XGBRFC)	0.830	0.800	0.850	0.650	0.840	0.820
	PEP-PRED^Na+^ (ET)	0.830	0.830	0.830	0.670	0.830	0.830
	PEP-PRED^Na+^ (RF)	0.830	0.810	0.860	0.670	0.850	0.830
	PEP-PRED^Na+^ (XGBC)	0.830	0.820	0.850	0.670	0.840	0.830
	PEP-PRED^Na+^ (LGBMC)	0.840	0.840	0.850	0.680	0.840	0.840
	MetaNaBP	**0.984**	**0.969**	**1.000**	**0.969**	**1.000**	**0.984**
Independent test	PEP-PRED^Na+^ (XGBRFC)	0.910	0.860	**0.960**	0.820	0.950	0.900
	PEP-PRED^Na+^ (ET)	0.910	0.900	0.920	0.820	0.920	0.910
	PEP-PRED^Na+^ (RF)	0.920	0.880	**0.960**	0.840	**0.960**	0.910
	PEP-PRED^Na+^ (XGBC)	0.900	0.860	0.940	0.800	0.930	0.890
	PEP-PRED^Na+^ (LGBMC)	0.890	0.860	0.920	0.780	0.910	0.880
	MetaNaBP	**0.948**	**0.979**	0.917	**0.898**	0.917	**0.946**

The best performance value for each performance metrics across different methods is highlighted in bold.

Compared with PEP-PRED^Na+^ (LGBMC), the ACC, SN, SP, F1, and MCC of our proposed method significantly improved by 14.42, 12.88, 15.00, 14.43, and 28.69%, respectively. The improved performance of MetaNaBP on the training dataset suggests that the multi-view feature fusion strategy used herein can advance the efficiency of model training. Furthermore, on the independent test dataset, MetaNaBP also achieved the best ACC, SN, F1, and MCC, which were 5.79, 11.92, 6.62, and 11.76% higher than those of PEP-PRED^Na+^ (LGBMC). Overall, MetaNaBP surpassed the existing method and provided a stable prediction performance, indicating its effectiveness and generalization ability.

### Interpretability of the MetaNaBP model

**Figure 6:**
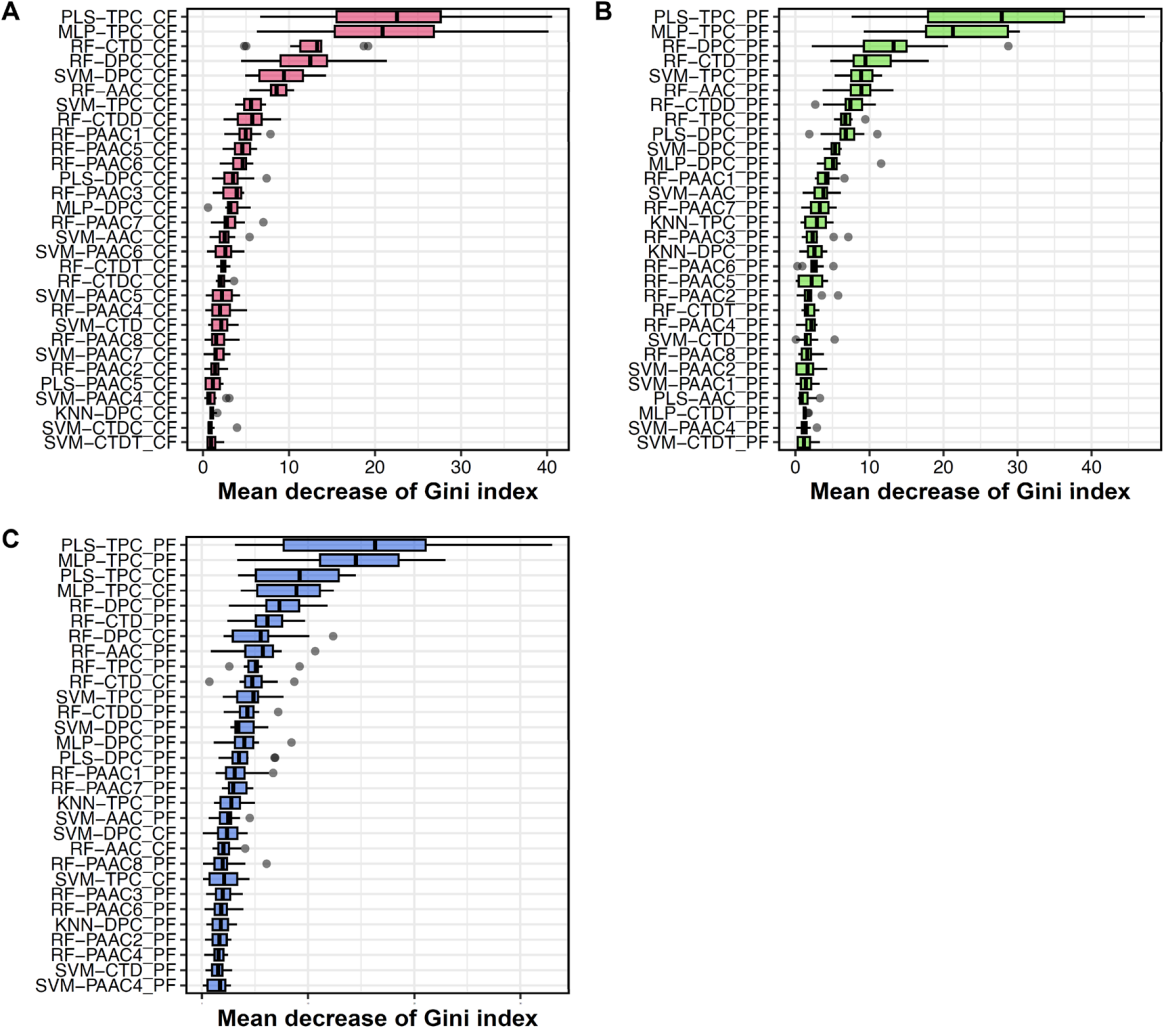
Feature importance of our feature representations (i.e., FS-CF (**A**), FS-PF (**B**), FS-PCF (**C**)). The feature having the largest value of mean decrease of Gini index (MDGI) is the most important one.

**Figure 7:**
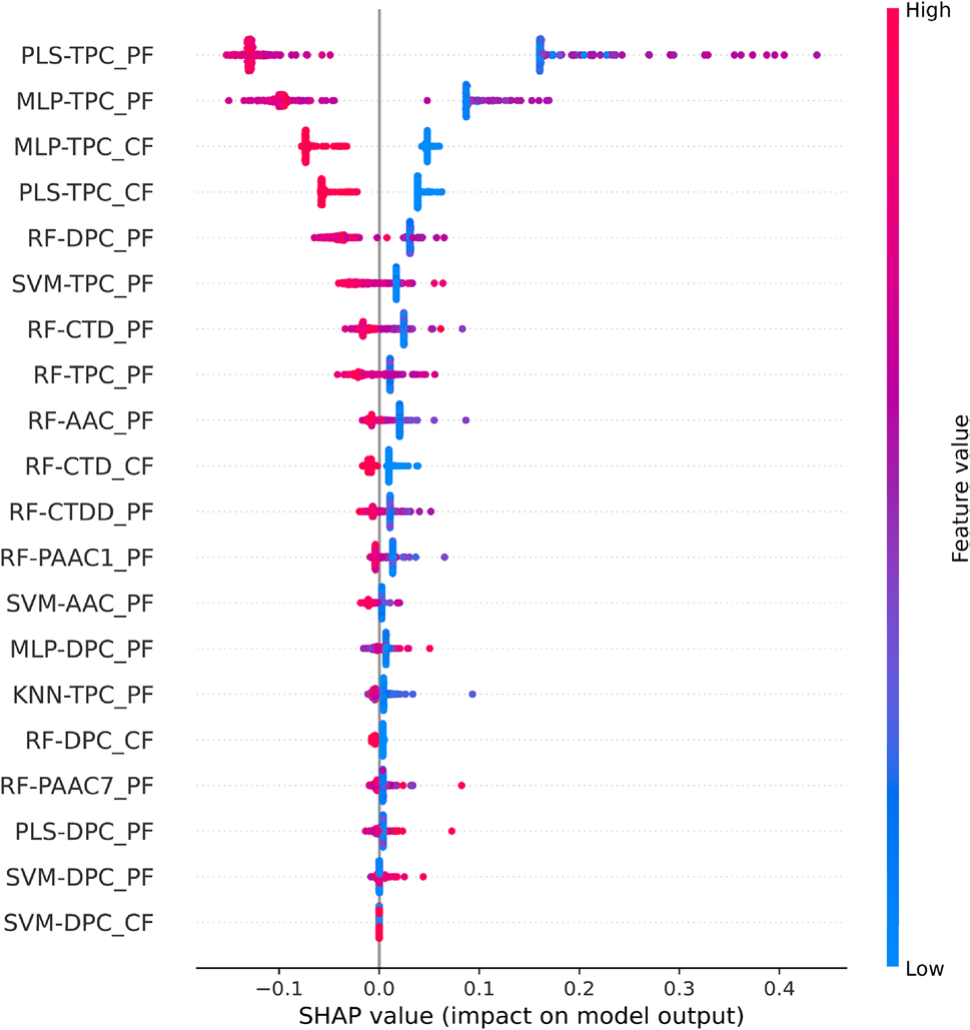
SHAP values of the 20 based-classifiers selected by the MetaNaBP.

**Figure 8:**
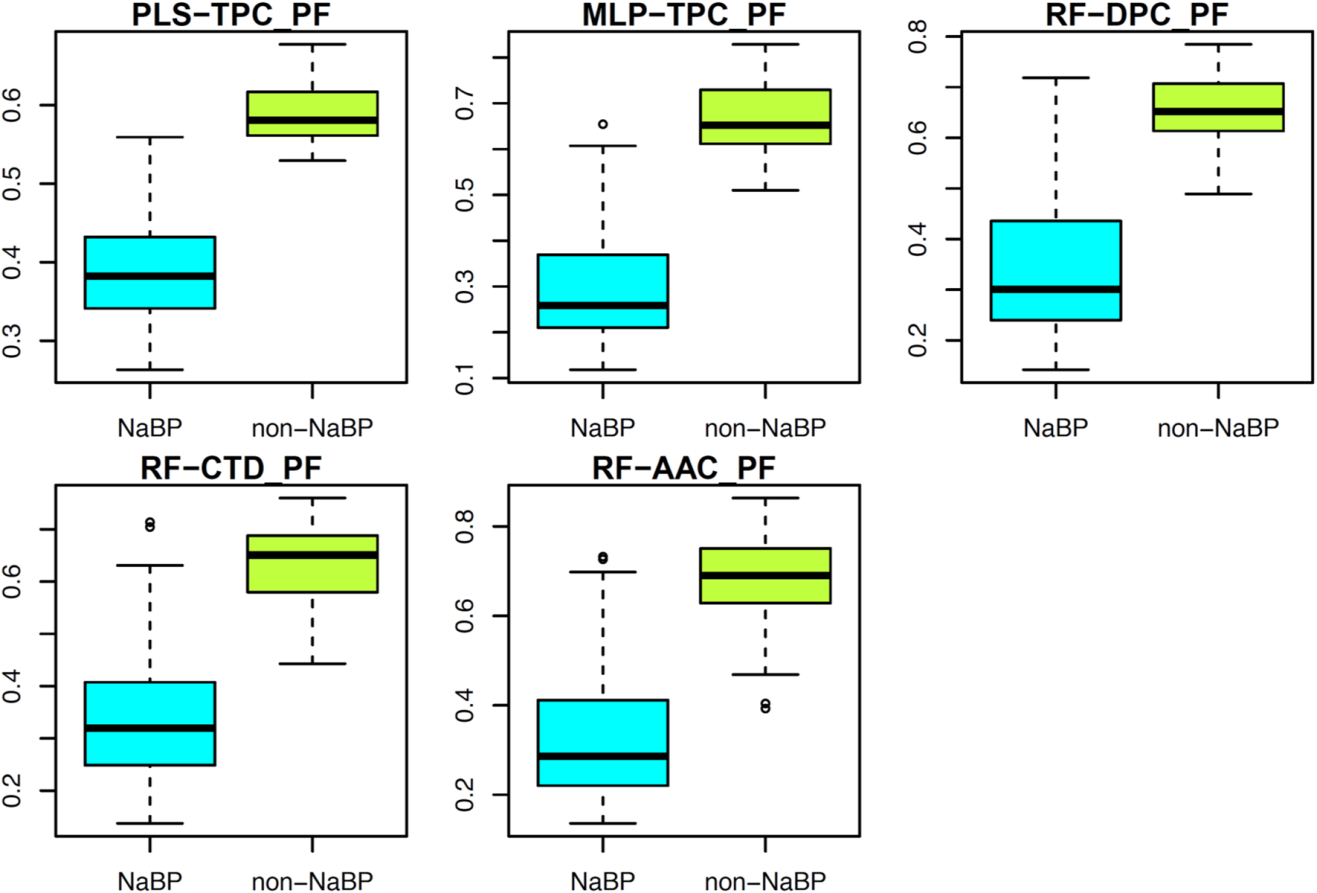
Boxplots of five important PFs selected by MDGI values on the training dataset for NaBPs and non-NaBPs.

In the feature selection process, we calculated the discriminative ability of each feature using MDGI. Figure 6 displays the ranks of feature importance for FS-CF, FS-PF, and FS-PCF. As mentioned above, because FS-PF and FS-PCF displayed better performance compared to FS-CF, we analyzed their impact to understand the prediction outputs of MetaNaBP. As seen in Fig. 6C, the top-ten informative features of FS-PCF include PLS-TPC_PF, MLP-TPC_PF, PLS-TPC_CF, MLP-TPC_CF, RF-DPC_PF, RF-CTD_PF, RF-DPC_CF, RF-AAC_PF, RF-TPC_PF, and RF-CTD_CF, with corresponding MDGI values of 15.64, 14.03, 9.22, 8.35, 7.30, 6.18, 5.54, 5.53, 5.08, and 4.88. In the meanwhile, PLS-TPC_PF, MLP-TPC_PF, RF-DPC_PF, RF-CTD_PF, and RF-AAC_PF were identified among the top-ten informative features for both FS-PF and FS-PCF (Fig. 6B,C). To reveal the directionality of these features, we employed the Shapley Additive explanation (SHAP) approach. Figure 7 shows that the values for these five highlighted features tend to be relatively low for most non-NaBPs and high for most NaBPs. This observation is further confirmed by the five boxplots illustrated in Fig. 8. It suggests that these five important features are beneficial for discriminating NaBPs from non-NaBPs and play a pivitol role in achieving the improved performance of MetaNaBP.

### Future perspective and direction of NaBP prediction models

Rapid and accurate identification of NaBPs based solely on sequence information is capable of enhancing the comprehension of the structure and function of targeted NaBPs at the molecular level. This understanding proves valuable for the screening and design of therapeutic drugs for pain. Specific NaBPs targeting the Nav channel have been proposed through computational design, followed by experimental validation. For instance, the computationally designed NaBPs (PTx2-3127 and PTx2-3258), inspired by ProTx-II from tarantula venom, effectively inhibit Nav1.7 activation in mouse and human sensory neurons^55,56^. At the cellular level, validation through a tetrazolium-based mouse neuroblastoma cell assay revealed that the NaBP candidate 'CcNT,' derived from the tentacle venom of the *Scyphozoa C. capillata*, specifically inhibits Na_v_ channels at receptor site 1^57^. In tissue and animal model experiments, rodents are commonly used for physiological and systemic validations of potential NaBP candidates from the computational design pipeline. For example, PTx2-3127 inhibits Na_v_1.7 and demonstrates efficacy in rat models of chronic and thermal pain when administered intrathecally^55^. Notably, cytotoxicity and cardiotoxicity assays are also required to avoid the undesirable side effects of NaBPs^58^. In addition to chronic and thermal pain experiments, inflammatory and neuropathic pain in rodent models can also be considered for the functional validation of NaBPs, similar to the case of μ-EPTX-Na1a from the venom of the Chinese cobra (*Naja atra*)^58^.

In the future work, we will incorporate our proposed ML framework with ordinary differential equation (ODE)-based theoretical modelling to provide insights into the dynamics, interactions, and effects of NaBPs on specific Nav targets, revealing regulatory mechanisms similar to studies on gene/protein signaling networks aimed at identifying therapeutic targets in diseases^59,60^. Additionally, advanced interaction predictive models based on deep learning methods could predict associations between NaBP candidates and specific Nav targets, mirroring the development of computational models identifying relationships between genetic markers and diseases^61–63^. Beyond bioactive peptides, non-coding RNAs (ncRNAs), such as MicroRNAs (miRNAs) and long non-coding RNAs (lncRNAs), also have potential as diverse channel blockers in neurons ^64–66^. In this context, a meta-learning approach could be applied alongside interaction predictive models for NaBPs and ncRNAs, aiming to understand their functions and identify related protein targets. This approach aligns with various deep learning methods that have utilized similar algorithms in molecular marker studies for other diseases^63,67–72^.

## Conclusions

In this study, we have developed a novel and high-accuracy computational model to accurately identify NaBPs, named MetaNaBP. To the best of our knowledge, MetaNaBP is the first meta-learning model designed for NaBPs prediction. MetaNaBP utilizes a wide range of sequence-based feature descriptors that cover multiple perspectives, inputting them to six powerful ML algorithms to construct several base-classifiers. These base-classifiers, in turn, provide useful feature representations. Simultaneously, these feature representations were optimized using a two-step feature selection method to identify the most effective feature set, which was then applied to develop the final meta-predictor. To validate the performance of MetaNaBP, we conducted a comparative analysis of our meta-learning model and its base-classifiers, along with an existing method. The results from both cross-validation and independent tests indicated that MetaNaBP outperformed its base-classifiers, underscoring the effectiveness of the meta-learning approach. Impressively, on the independent test dataset, MetaNaBP was capable of increasing MCC of 0.820 to 0.898, ACC of 0.890 to 0.948, SN of 0.900 to 0.979. This indicates that MetaNaBP could be a useful tool to precisely identify NaBPs. Anticipating MetaNaBP to be a useful tool, we envision it playing a crucial role in drug discovery and development by screening and identifying potential NaBPs. Although MetaNaBP achieved high-accuracy performance in NaBP prediction, we still have a few aims to enhance its overall performance in our future work. Firstly, we aim to employ other types of feature encoding schemes, such as Word2vec and one-hot encoding methods^73^. Secondly, we plan to integrate our proposed model with powerful deep learning methods, such as convolutional neural networks and long short-term memory networks^74^. Lastly, we intend to establish a web server to directly perform NaBP prediction based solely on the primary sequence.

### Supplementary Information

Supplementary Information.

## Data Availability

All the data used in this study are available at https://github.com/Shoombuatong/MetaNaBP.
