# Supplementary material for: Leveraging a meta-learning approach to advance the accuracy of Nav blocking peptides prediction
Source: Sci Rep. 2024 Feb 23;14:4463. doi: 10.1038/s41598-024-55160-z (PMC10891130; doi:10.1038/s41598-024-55160-z)
Supplement: Supplementary file 1 — Supplementary Information. [file 41598_2024_55160_MOESM1_ESM.docx]

## **Supplementary Figure**


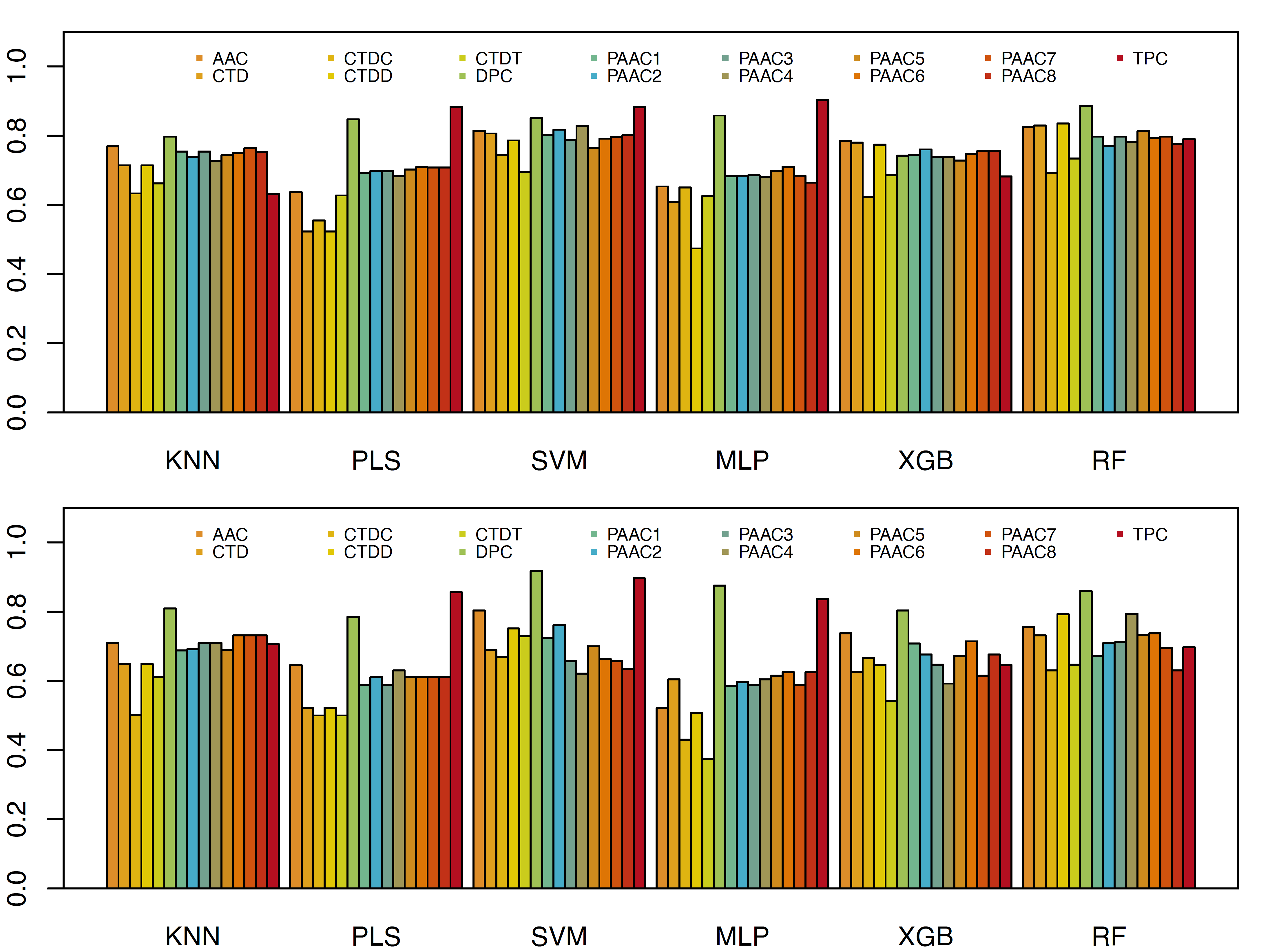


**B**

**A**

**Figure S1.** MCC values of various ML classifiers as evaluated using the training (**A**) and independent (**B**) datasets.

## **Supplementary Table**

**Table S1.** Lists of physicochemical properties and groups of amino acids.

| **Physicochemical property** | **Group 1** | **Group 2** | **Group 3** |
| --- | --- | --- | --- |
| Hydrophobicity | CFILMVW | AGHPSTY | DEKNQR |
| Polarity | CFILMVWY | AGPST | DEHKNQR |
| Normalized Van Der Waals Volume | ACDGPST | EILNQV | FHKMRWYVWY |
| Polarizability | ADGST | CEILNPQV | FHKMRWY |
| Charge | ACFGHILMNPQSTVWY | DE | KR |
| Solvent Solubility | ACFGILVW | HMPSTY | DEKNRQ |
| Secondary Structure | DGNPS | AEHKLMQR | CFITVWY |

**Table S2.** Information of parameter settings for six ML methods used in this study.

| **Method** | **Parameters** | **Search space** |
| --- | --- | --- |
| PLS | *ncomp* | [1, 2, 3, 4, 5] |
| KNN | *k* | [1, 3, 5, 7, 9] |
| MLP | *size* | [1, 3, 5, 7, 9] |
| SVM | *C* | [0.25, 0.5, 1, 2, 4] |
| RF | *mtry* | [5, 7, 10, 15, 20, 25] |
|  | *ntree* | [20, 50, 100, 200, 300] |
| XGB | *nrounds* | [20, 50, 100, 200, 300] |
|  | *max_depth* | [3, 5, 7, 9, 10] |
|  | *eta* | [0.1, 0.2, 0.3] |
|  | *gamma* | 0 |
|  | *colsample_bytree* | 0.7 |
|  | *min_child_weight* | 5 |
|  | *subsample* | 0.5 |

**Table S3** Cross-validation results of 90 ML classifiers developed using six ML algorithms and fifteen types of feature descriptors.

| **Descriptor** | **ML algorithm** | **ACC** | **SN** | **SP** | **MCC** | **AUC** |
| --- | --- | --- | --- | --- | --- | --- |
| AAC | KNN | 0.884 | 0.901 | 0.867 | 0.769 | 0.932 |
|  | PLS | 0.818 | 0.828 | 0.809 | 0.637 | 0.882 |
|  | SVM | 0.905 | 0.865 | 0.947 | 0.814 | 0.975 |
|  | MLP | 0.826 | 0.813 | 0.840 | 0.653 | 0.889 |
|  | XGB | 0.892 | 0.870 | 0.915 | 0.785 | 0.945 |
|  | RF | 0.911 | 0.865 | 0.957 | 0.825 | 0.976 |
| CTD | KNN | 0.855 | 0.906 | 0.803 | 0.714 | 0.881 |
|  | PLS | 0.761 | 0.807 | 0.713 | 0.523 | 0.812 |
|  | SVM | 0.903 | 0.880 | 0.926 | 0.806 | 0.967 |
|  | MLP | 0.803 | 0.859 | 0.745 | 0.608 | 0.862 |
|  | XGB | 0.889 | 0.870 | 0.910 | 0.780 | 0.947 |
|  | RF | 0.913 | 0.875 | 0.952 | 0.829 | 0.970 |
| CTDC | KNN | 0.816 | 0.849 | 0.782 | 0.633 | 0.868 |
|  | PLS | 0.776 | 0.828 | 0.723 | 0.555 | 0.861 |
|  | SVM | 0.871 | 0.854 | 0.888 | 0.743 | 0.935 |
|  | MLP | 0.821 | 0.750 | 0.894 | 0.650 | 0.872 |
|  | XGB | 0.811 | 0.786 | 0.835 | 0.622 | 0.899 |
|  | RF | 0.845 | 0.807 | 0.883 | 0.692 | 0.931 |
| CTDD | KNN | 0.855 | 0.906 | 0.803 | 0.714 | 0.881 |
|  | PLS | 0.761 | 0.807 | 0.713 | 0.523 | 0.812 |
|  | SVM | 0.892 | 0.865 | 0.920 | 0.786 | 0.951 |
|  | MLP | 0.737 | 0.734 | 0.739 | 0.474 | 0.856 |
|  | XGB | 0.887 | 0.885 | 0.888 | 0.774 | 0.949 |
|  | RF | 0.916 | 0.875 | 0.957 | 0.835 | 0.971 |
| CTDT | KNN | 0.829 | 0.891 | 0.766 | 0.662 | 0.875 |
|  | PLS | 0.813 | 0.839 | 0.787 | 0.627 | 0.893 |
|  | SVM | 0.847 | 0.833 | 0.862 | 0.695 | 0.930 |
|  | MLP | 0.813 | 0.807 | 0.819 | 0.626 | 0.903 |
|  | XGB | 0.842 | 0.828 | 0.856 | 0.685 | 0.916 |
|  | RF | 0.866 | 0.828 | 0.904 | 0.734 | 0.932 |
| DPC | KNN | 0.892 | 0.807 | 0.979 | 0.797 | 0.916 |
|  | PLS | 0.924 | 0.922 | 0.926 | 0.847 | 0.966 |
|  | SVM | 0.924 | 0.880 | 0.968 | 0.851 | 0.981 |
|  | MLP | 0.929 | 0.927 | 0.931 | 0.858 | 0.971 |
|  | XGB | 0.871 | 0.859 | 0.883 | 0.742 | 0.942 |
|  | RF | 0.939 | 0.880 | 1.000 | 0.886 | 0.977 |
| PAAC (λ = 1) | KNN | 0.876 | 0.854 | 0.899 | 0.754 | 0.935 |
|  | PLS | 0.845 | 0.797 | 0.894 | 0.693 | 0.905 |
|  | SVM | 0.900 | 0.875 | 0.926 | 0.801 | 0.952 |
|  | MLP | 0.839 | 0.792 | 0.888 | 0.683 | 0.900 |
|  | XGB | 0.871 | 0.849 | 0.894 | 0.743 | 0.929 |
|  | RF | 0.897 | 0.859 | 0.936 | 0.797 | 0.962 |
| PAAC (λ = 2) | KNN | 0.868 | 0.849 | 0.888 | 0.738 | 0.932 |
|  | PLS | 0.847 | 0.802 | 0.894 | 0.698 | 0.900 |
|  | SVM | 0.908 | 0.885 | 0.931 | 0.817 | 0.951 |
|  | MLP | 0.839 | 0.781 | 0.899 | 0.684 | 0.900 |
|  | XGB | 0.879 | 0.849 | 0.910 | 0.760 | 0.933 |
|  | RF | 0.884 | 0.854 | 0.915 | 0.770 | 0.965 |
| PAAC (λ = 3) | KNN | 0.876 | 0.854 | 0.899 | 0.754 | 0.928 |
|  | PLS | 0.847 | 0.807 | 0.888 | 0.697 | 0.902 |
|  | SVM | 0.892 | 0.849 | 0.936 | 0.788 | 0.948 |
|  | MLP | 0.842 | 0.828 | 0.856 | 0.685 | 0.899 |
|  | XGB | 0.868 | 0.839 | 0.899 | 0.738 | 0.924 |
|  | RF | 0.897 | 0.859 | 0.936 | 0.797 | 0.961 |
| PAAC (λ = 4) | KNN | 0.863 | 0.849 | 0.878 | 0.727 | 0.931 |
|  | PLS | 0.839 | 0.792 | 0.888 | 0.683 | 0.899 |
|  | SVM | 0.913 | 0.880 | 0.947 | 0.828 | 0.949 |
|  | MLP | 0.839 | 0.818 | 0.862 | 0.680 | 0.905 |
|  | XGB | 0.868 | 0.849 | 0.888 | 0.738 | 0.930 |
|  | RF | 0.889 | 0.854 | 0.926 | 0.781 | 0.963 |
| PAAC(λ = 5) | KNN | 0.871 | 0.849 | 0.894 | 0.743 | 0.929 |
|  | PLS | 0.850 | 0.813 | 0.888 | 0.702 | 0.903 |
|  | SVM | 0.882 | 0.854 | 0.910 | 0.765 | 0.943 |
|  | MLP | 0.847 | 0.802 | 0.894 | 0.698 | 0.899 |
|  | XGB | 0.863 | 0.828 | 0.899 | 0.728 | 0.939 |
|  | RF | 0.905 | 0.870 | 0.941 | 0.813 | 0.967 |
| PAAC(λ = 6) | KNN | 0.874 | 0.849 | 0.899 | 0.749 | 0.928 |
|  | PLS | 0.853 | 0.807 | 0.899 | 0.709 | 0.903 |
|  | SVM | 0.895 | 0.870 | 0.920 | 0.791 | 0.940 |
|  | MLP | 0.855 | 0.859 | 0.851 | 0.710 | 0.899 |
|  | XGB | 0.874 | 0.870 | 0.878 | 0.747 | 0.924 |
|  | RF | 0.895 | 0.849 | 0.941 | 0.793 | 0.969 |
| PAAC (λ = 7) | KNN | 0.882 | 0.865 | 0.899 | 0.764 | 0.926 |
|  | PLS | 0.853 | 0.813 | 0.894 | 0.708 | 0.903 |
|  | SVM | 0.897 | 0.875 | 0.920 | 0.796 | 0.945 |
|  | MLP | 0.842 | 0.844 | 0.840 | 0.684 | 0.901 |
|  | XGB | 0.876 | 0.839 | 0.915 | 0.755 | 0.931 |
|  | RF | 0.897 | 0.865 | 0.931 | 0.797 | 0.965 |
| PAAC (λ = 8) | KNN | 0.876 | 0.859 | 0.894 | 0.753 | 0.923 |
|  | PLS | 0.853 | 0.813 | 0.894 | 0.708 | 0.904 |
|  | SVM | 0.900 | 0.875 | 0.926 | 0.801 | 0.956 |
|  | MLP | 0.832 | 0.813 | 0.851 | 0.664 | 0.900 |
|  | XGB | 0.876 | 0.839 | 0.915 | 0.755 | 0.938 |
|  | RF | 0.887 | 0.849 | 0.926 | 0.776 | 0.961 |
| TPC | KNN | 0.784 | 0.573 | 1.000 | 0.632 | 0.784 |
|  | PLS | 0.939 | 0.896 | 0.984 | 0.883 | 0.989 |
|  | SVM | 0.939 | 0.901 | 0.979 | 0.882 | 0.988 |
|  | MLP | 0.950 | 0.917 | 0.984 | 0.902 | 0.988 |
|  | XGB | 0.829 | 0.703 | 0.957 | 0.682 | 0.875 |
|  | RF | 0.884 | 0.771 | 1.000 | 0.790 | 0.971 |

**Table S4** Independent test results of 90 ML classifiers developed using six ML algorithms and fifteen types of feature descriptors.

| **Descriptor** | **ML algorithm** | **ACC** | **SN** | **SP** | **MCC** | **AUC** |
| --- | --- | --- | --- | --- | --- | --- |
| AAC | KNN | 0.854 | 0.833 | 0.875 | 0.709 | 0.894 |
|  | PLS | 0.823 | 0.813 | 0.833 | 0.646 | 0.877 |
|  | SVM | 0.896 | 0.813 | 0.979 | 0.803 | 0.966 |
|  | MLP | 0.760 | 0.750 | 0.771 | 0.521 | 0.866 |
|  | XGB | 0.865 | 0.792 | 0.938 | 0.737 | 0.947 |
|  | RF | 0.875 | 0.813 | 0.938 | 0.756 | 0.934 |
| CTD | KNN | 0.823 | 0.875 | 0.771 | 0.649 | 0.842 |
|  | PLS | 0.760 | 0.792 | 0.729 | 0.522 | 0.782 |
|  | SVM | 0.844 | 0.813 | 0.875 | 0.689 | 0.929 |
|  | MLP | 0.802 | 0.792 | 0.813 | 0.604 | 0.867 |
|  | XGB | 0.813 | 0.792 | 0.833 | 0.626 | 0.915 |
|  | RF | 0.865 | 0.833 | 0.896 | 0.731 | 0.948 |
| CTDC | KNN | 0.750 | 0.708 | 0.792 | 0.502 | 0.791 |
|  | PLS | 0.750 | 0.771 | 0.729 | 0.500 | 0.853 |
|  | SVM | 0.833 | 0.792 | 0.875 | 0.669 | 0.926 |
|  | MLP | 0.708 | 0.833 | 0.583 | 0.430 | 0.837 |
|  | XGB | 0.833 | 0.813 | 0.854 | 0.667 | 0.905 |
|  | RF | 0.813 | 0.750 | 0.875 | 0.630 | 0.937 |
| CTDD | KNN | 0.823 | 0.875 | 0.771 | 0.649 | 0.842 |
|  | PLS | 0.760 | 0.792 | 0.729 | 0.522 | 0.782 |
|  | SVM | 0.875 | 0.854 | 0.896 | 0.751 | 0.927 |
|  | MLP | 0.750 | 0.667 | 0.833 | 0.507 | 0.842 |
|  | XGB | 0.823 | 0.833 | 0.813 | 0.646 | 0.929 |
|  | RF | 0.896 | 0.917 | 0.875 | 0.792 | 0.941 |
| CTDT | KNN | 0.802 | 0.875 | 0.729 | 0.611 | 0.862 |
|  | PLS | 0.750 | 0.750 | 0.750 | 0.500 | 0.796 |
|  | SVM | 0.865 | 0.854 | 0.875 | 0.729 | 0.929 |
|  | MLP | 0.688 | 0.708 | 0.667 | 0.375 | 0.759 |
|  | XGB | 0.771 | 0.750 | 0.792 | 0.542 | 0.860 |
|  | RF | 0.823 | 0.792 | 0.854 | 0.647 | 0.895 |
| DPC | KNN | 0.896 | 0.792 | 1.000 | 0.809 | 0.852 |
|  | PLS | 0.885 | 0.792 | 0.979 | 0.785 | 0.964 |
|  | SVM | 0.958 | 0.938 | 0.979 | 0.917 | 0.988 |
|  | MLP | 0.938 | 0.938 | 0.938 | 0.875 | 0.973 |
|  | XGB | 0.896 | 0.813 | 0.979 | 0.803 | 0.946 |
|  | RF | 0.927 | 0.875 | 0.979 | 0.859 | 0.984 |
| PAAC (λ = 1) | KNN | 0.844 | 0.833 | 0.854 | 0.688 | 0.893 |
|  | PLS | 0.792 | 0.729 | 0.854 | 0.588 | 0.887 |
|  | SVM | 0.854 | 0.750 | 0.958 | 0.724 | 0.959 |
|  | MLP | 0.792 | 0.771 | 0.813 | 0.584 | 0.889 |
|  | XGB | 0.854 | 0.854 | 0.854 | 0.708 | 0.923 |
|  | RF | 0.833 | 0.771 | 0.896 | 0.672 | 0.935 |
| PAAC (λ = 2) | KNN | 0.844 | 0.792 | 0.896 | 0.691 | 0.905 |
|  | PLS | 0.802 | 0.729 | 0.875 | 0.611 | 0.874 |
|  | SVM | 0.875 | 0.792 | 0.958 | 0.761 | 0.961 |
|  | MLP | 0.792 | 0.688 | 0.896 | 0.596 | 0.890 |
|  | XGB | 0.833 | 0.750 | 0.917 | 0.676 | 0.924 |
|  | RF | 0.854 | 0.833 | 0.875 | 0.709 | 0.931 |
| PAAC (λ = 3) | KNN | 0.854 | 0.833 | 0.875 | 0.709 | 0.881 |
|  | PLS | 0.792 | 0.729 | 0.854 | 0.588 | 0.885 |
|  | SVM | 0.823 | 0.729 | 0.917 | 0.657 | 0.952 |
|  | MLP | 0.792 | 0.729 | 0.854 | 0.588 | 0.893 |
|  | XGB | 0.823 | 0.792 | 0.854 | 0.647 | 0.915 |
|  | RF | 0.854 | 0.813 | 0.896 | 0.711 | 0.934 |
| PAAC (λ = 4) | KNN | 0.854 | 0.833 | 0.875 | 0.709 | 0.870 |
|  | PLS | 0.813 | 0.750 | 0.875 | 0.630 | 0.874 |
|  | SVM | 0.802 | 0.688 | 0.917 | 0.621 | 0.946 |
|  | MLP | 0.802 | 0.792 | 0.813 | 0.604 | 0.888 |
|  | XGB | 0.792 | 0.708 | 0.875 | 0.592 | 0.892 |
|  | RF | 0.896 | 0.854 | 0.938 | 0.794 | 0.945 |
| PAAC(λ = 5) | KNN | 0.844 | 0.813 | 0.875 | 0.689 | 0.880 |
|  | PLS | 0.802 | 0.729 | 0.875 | 0.611 | 0.885 |
|  | SVM | 0.844 | 0.750 | 0.938 | 0.700 | 0.956 |
|  | MLP | 0.802 | 0.708 | 0.896 | 0.615 | 0.896 |
|  | XGB | 0.833 | 0.771 | 0.896 | 0.672 | 0.918 |
|  | RF | 0.865 | 0.813 | 0.917 | 0.733 | 0.932 |
| PAAC(λ = 6) | KNN | 0.865 | 0.833 | 0.896 | 0.731 | 0.870 |
|  | PLS | 0.802 | 0.729 | 0.875 | 0.611 | 0.884 |
|  | SVM | 0.823 | 0.708 | 0.938 | 0.663 | 0.944 |
|  | MLP | 0.813 | 0.813 | 0.813 | 0.625 | 0.888 |
|  | XGB | 0.854 | 0.792 | 0.917 | 0.714 | 0.911 |
|  | RF | 0.865 | 0.792 | 0.938 | 0.737 | 0.939 |
| PAAC (λ = 7) | KNN | 0.865 | 0.833 | 0.896 | 0.731 | 0.894 |
|  | PLS | 0.802 | 0.729 | 0.875 | 0.611 | 0.883 |
|  | SVM | 0.823 | 0.729 | 0.917 | 0.657 | 0.946 |
|  | MLP | 0.792 | 0.729 | 0.854 | 0.588 | 0.887 |
|  | XGB | 0.802 | 0.708 | 0.896 | 0.615 | 0.920 |
|  | RF | 0.844 | 0.771 | 0.917 | 0.695 | 0.938 |
| PAAC (λ = 8) | KNN | 0.865 | 0.833 | 0.896 | 0.731 | 0.881 |
|  | PLS | 0.802 | 0.729 | 0.875 | 0.611 | 0.885 |
|  | SVM | 0.813 | 0.729 | 0.896 | 0.634 | 0.940 |
|  | MLP | 0.813 | 0.813 | 0.813 | 0.625 | 0.886 |
|  | XGB | 0.833 | 0.750 | 0.917 | 0.676 | 0.915 |
|  | RF | 0.813 | 0.750 | 0.875 | 0.630 | 0.921 |
| TPC | KNN | 0.833 | 0.667 | 1.000 | 0.707 | 0.771 |
|  | PLS | 0.927 | 0.896 | 0.958 | 0.856 | 0.989 |
|  | SVM | 0.948 | 0.938 | 0.958 | 0.896 | 0.985 |
|  | MLP | 0.917 | 0.875 | 0.958 | 0.836 | 0.987 |
|  | XGB | 0.813 | 0.688 | 0.938 | 0.645 | 0.893 |
|  | RF | 0.833 | 0.688 | 0.979 | 0.697 | 0.974 |

**Table S5** Cross-validation and independent test results of top-five ML classifiers having the highest cross-validation MCC.

| **Evaluation strategy** | **Method** | **ACC** | **SN** | **SP** | **MCC** | **AUC** |
| --- | --- | --- | --- | --- | --- | --- |
| Cross-validation | MLP-TPC | 0.950 | 0.917 | 0.984 | 0.902 | 0.988 |
|  | RF-DPC | 0.939 | 0.880 | 1.000 | 0.886 | 0.977 |
|  | PLS-TPC | 0.939 | 0.896 | 0.984 | 0.883 | 0.989 |
|  | SVM-TPC | 0.939 | 0.901 | 0.979 | 0.882 | 0.988 |
|  | MLP-DPC | 0.929 | 0.927 | 0.931 | 0.858 | 0.971 |
| Independent test | MLP-TPC | 0.917 | 0.875 | 0.958 | 0.836 | 0.987 |
|  | RF-DPC | 0.927 | 0.875 | 0.979 | 0.859 | 0.984 |
|  | PLS-TPC | 0.927 | 0.896 | 0.958 | 0.856 | 0.989 |
|  | SVM-TPC | 0.948 | 0.938 | 0.958 | 0.896 | 0.985 |
|  | MLP-DPC | 0.938 | 0.938 | 0.938 | 0.875 | 0.973 |

**Table S6** Average cross-validation results of each feature encoding over six ML methods.

| **Descriptor** | **ACC** | **SN** | **SP** | **MCC** | **AUC** |
| --- | --- | --- | --- | --- | --- |
| AAC | 0.911 | 0.865 | 0.957 | 0.825 | 0.976 |
| CTD | 0.913 | 0.875 | 0.952 | 0.829 | 0.970 |
| CTDC | 0.845 | 0.807 | 0.883 | 0.692 | 0.931 |
| CTDD | 0.916 | 0.875 | 0.957 | 0.835 | 0.971 |
| CTDT | 0.866 | 0.828 | 0.904 | 0.734 | 0.932 |
| DPC | 0.939 | 0.880 | 1.000 | 0.886 | 0.977 |
| PAAC(λ = 1) | 0.897 | 0.859 | 0.936 | 0.797 | 0.962 |
| PAAC(λ = 2) | 0.884 | 0.854 | 0.915 | 0.770 | 0.965 |
| PAAC(λ = 3) | 0.897 | 0.859 | 0.936 | 0.797 | 0.961 |
| PAAC(λ = 4) | 0.889 | 0.854 | 0.926 | 0.781 | 0.963 |
| PAAC(λ = 5) | 0.905 | 0.870 | 0.941 | 0.813 | 0.967 |
| PAAC(λ = 6) | 0.895 | 0.849 | 0.941 | 0.793 | 0.969 |
| PAAC(λ = 7) | 0.897 | 0.865 | 0.931 | 0.797 | 0.965 |
| PAAC(λ = 8) | 0.887 | 0.849 | 0.926 | 0.776 | 0.961 |
| TPC | 0.884 | 0.771 | 1.000 | 0.790 | 0.971 |
| FS-PCF (Our feature) | 0.987 | 0.974 | 1.000 | 0.974 | 0.991 |

**Table S7** Performance comparison of our feature (FS-PCF) and conventional feature descriptors on the training dataset

| **Feature** | **ACC** | **SN** | **SP** | **MCC** | **AUC** |
| --- | --- | --- | --- | --- | --- |
| AAC | 0.911 | 0.865 | 0.957 | 0.825 | 0.976 |
| CTD | 0.913 | 0.875 | 0.952 | 0.829 | 0.970 |
| CTDC | 0.845 | 0.807 | 0.883 | 0.692 | 0.931 |
| CTDD | 0.916 | 0.875 | 0.957 | 0.835 | 0.971 |
| CTDT | 0.866 | 0.828 | 0.904 | 0.734 | 0.932 |
| DPC | 0.939 | 0.880 | 1.000 | 0.886 | 0.977 |
| PAAC(λ = 1) | 0.897 | 0.859 | 0.936 | 0.797 | 0.962 |
| PAAC(λ = 2) | 0.884 | 0.854 | 0.915 | 0.770 | 0.965 |
| PAAC(λ = 3) | 0.897 | 0.859 | 0.936 | 0.797 | 0.961 |
| PAAC(λ = 4) | 0.889 | 0.854 | 0.926 | 0.781 | 0.963 |
| PAAC(λ = 5) | 0.905 | 0.870 | 0.941 | 0.813 | 0.967 |
| PAAC(λ = 6) | 0.895 | 0.849 | 0.941 | 0.793 | 0.969 |
| PAAC(λ = 7) | 0.897 | 0.865 | 0.931 | 0.797 | 0.965 |
| PAAC(λ = 8) | 0.887 | 0.849 | 0.926 | 0.776 | 0.961 |
| TPC | 0.884 | 0.771 | 1.000 | 0.790 | 0.971 |
| FS-PCF | 0.987 | 0.974 | 1.000 | 0.974 | 0.991 |
